# Long range infrasound monitoring of Etna volcano

**DOI:** 10.1038/s41598-019-54468-5

**Published:** 2019-11-29

**Authors:** E. Marchetti, M. Ripepe, P. Campus, A. Le Pichon, J. Vergoz, G. Lacanna, P. Mialle, P. Héreil, P. Husson

**Affiliations:** 1 University of Firenze, Department of Earth Sciences, via G. La Pira, 4, 50121 Firenze, Italy; 2 CEA, DAM, DIF, F-91297, Arpajon, 91680 France; 3 CTBTO, IDC, Vienna International Center, 1400 Vienna, Austria; 4 Meteo France, VAAC Toulouse, 42 Av. Coriolis, 31057 Toulouse, Cedex 1 France

**Keywords:** Natural hazards, Volcanology

## Abstract

Among ground-based volcano monitoring techniques, infrasound is the only one capable of detecting explosive eruptions from distances of thousands of kilometers. We show how infrasound array analysis, using acoustic amplitude and detection persistency, allows automatic, near-real-time identification of eruptions of Etna volcano (Italy), for stations at distances greater than 500 km. A semi-empirical attenuation relation is applied to recover the pressure time history at the source using infrasound recorded at global scale (>500 km). An infrasound parameter (IP), defined as the product between the number of detections, filtered for the expected back-azimuth of Etna volcano, and range corrected amplitude, is compared with the explosive activity at Etna volcano that was associated with aviation color code RED warnings. This shows that, during favourable propagation conditions, global arrays are capable of identifying explosive activity of Etna 87% of the period of analysis without negative false alerts. Events are typically not detected during unfavourable propagation conditions, thus resulting in a time variable efficiency of the system. We suggest that infrasound monitoring on a global scale can provide timely input for Volcanic Ash Advisory Centres (VAAC) even when a latency of ~1 hour, due to propagation time, is considered. The results highlight the capability of infrasound for near-real-time volcano monitoring at a regional and global scale.

## Introduction

Since 1950, our society experienced an escalation in communications and transport, becoming more vulnerable to the effects of large atmospheric ash dispersion during volcanic eruptions both at a local and on a global scale.

Over the recent decades, volcanic ash encounters by civil aviation have come very close to disasters on several occasions. Volcanic ash entering the aircraft’s jet turbine can cause ignition flameout and engine shutdown, with the melting temperature of silicate ash being lower than the engine operating temperature. Damages have been reported both for short duration flights within thick ash clouds (with ash concentrations >2 g/m^3^) and for prolonged exposures to dilute ash^1,2^.

Between 1953 and 2009, 94 aeroplane incidents have been confirmed as being due to ash encounters^3,4^ and in 9 cases caused engine shutdown during flight^3^. Most of the damaging encounters have occurred within 24 hours from the eruption onset or at relatively close distance (<1000 km) from the volcano^3^. Most encounters have occurred during moderate-sized eruptions, having a Volcanic Explosivity Index (VEI)^5^, spanning between two and three^3^. These types of eruptions (VEI > = 2) are expected to occur, globally, quite frequently (approximately 20 times/year)^6^, and are capable of generating ash plumes reaching the cruise altitude of commercial aircrafts. These mostly operate at Flight Levels between FL 200 (20,000 feet) and FL 350 (35,000 feet), ranging between ~6,100 and ~10,600 m of altitude.

Considering the number of active volcanoes in areas of dense commercial flight routes, it is clear that aviation is constantly subject to the big threat of a volcanic eruption. As a consequence, in 1990, the International Civil Aviation Organization (ICAO) established the Volcanic Ash Advisory Centres (VAAC), to mitigate the risk of volcanic eruptions on commercial flights^7^. VAACs liaise between meteorologists, vulcanologists and the aviation community. Whenever a volcanic eruption occurs, they gather all the available information from volcano observatories, satellite images and pilot reports and run dispersion models of volcanic plumes^8^. They eventually issue a Volcanic Ash Advisory (VAA), that provides information on the amount of ash and the dispersal forecasts.

A straightforward example is the 2010 eruption of Eyjafjallajökull volcano. The volcano generated an ash plume with a height of less than 9 km, but for about one week it created the highest level of air travel disruption, seen in Europe, since the Second World War. The effects of such a relatively small eruption (VEI 3/4) lead the civil aviation authority to ask for a prompt notification of volcanic eruptions and a quantitative estimation of plume evolution and atmospheric ash concentration^8^.

The quality and accuracy of the plume extension modeling, and ash dispersal forecasts, is strongly dependent on eruptive source parameters, such as the eruption onset time, location, and the mass of material erupted^8^. Unfortunately, these parameters are not generally available and most of the active volcanoes worldwide are not monitored with local networks.

Recently, the explosive eruption of Bogoslof volcano, in Alaska, was monitored by using multiple geophysical parameters (seismic/infrasound/lightning detection system and satellites) collected by distal stations^9^. Notification and warnings of eruptions, that produced ash clouds exceeding 7.5 km, were delivered by the 24/7 monitoring center. However, the distribution and capabilities of Volcano Observatories is very inhomogeneous worldwide. This makes efforts of automatic detections of ongoing volcanic eruptions, at various ranges, extremely valuable.

During the last decade, experiments on automatic detections and notification of volcanic eruptions with infrasound arrays were performed in South America^10^ and in Italy^11,12^. The Acoustic Surveillance for Hazardous Eruptions (ASHE) project^10,13^ delivered automatic notifications to the VAACs on the onset, and end times, of large explosive eruptions of the Tungurahua volcano (Ecuador)^14^.

More recently, a fully automated and operational warning system based on local (<6 km) infrasound array data was developed for Etna volcano in Italy. During a 10 year-long period, the system issued pre-alert notifications, preceding on average by 74 minutes, of the occurrence of the eruption with a reliability rate of 96.5% and without negative false alerts^12^.

Many studies have demonstrated the efficacy of infrasound to detect signals produced during volcanic eruptions at large (>several hundreds of km) distances^15–21^. Thanks to the limited attenuation of infrasonic waves travelling within atmospheric waveguides^22^, infrasound can propagate at long ranges when favourable propagation conditions exist. The eruptive time history can be tracked with greater temporal resolution compared to the information retrieved from satellite data^18^.

However, propagation in the atmosphere can also negatively affect signal detectability in upwind situation. Long-range infrasound detections are not necessarily reflecting the pressure time history at the source^23^. The maximum distance of detected infrasound appears to increase with the plume height^17^, which seems to indicate that in general, higher energy eruptions can be recorded at larger distances. Nevertheless, there are many examples of signals not-detected, even for high-energy events, as well as examples showing a complex time dependence between plume height and infrasonic amplitude^24^.

In this work, we investigate the potential of infrasound for detecting and monitoring explosive eruptions from Etna volcano (Italy), at long range, by using three large-aperture arrays deployed at a source-to-receiver distance of 600–1000 km from Mt. Etna volcano. Data from a local array are used for comparison and validation.

We show how, once propagation in the atmosphere is considered, acoustic pressure at the receiver can be converted in pressure at the source with a great degree of reliability. In addition, we assess the efficiency of notification provided by remote arrays with respect to the VAAs by the Toulouse VAAC, using information provided by the local monitoring agency.

## Results

**Figure 1:**
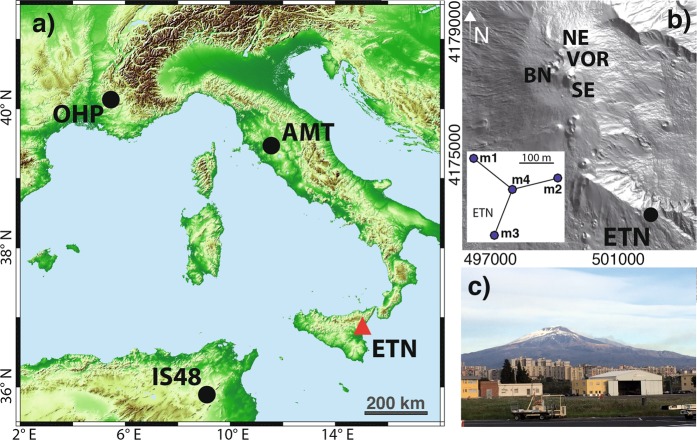
(**a**) Map of the Eastern Mediterranean showing the position of Etna Volcano (red triangle) in Sicily, Italy, and remote infrasound arrays (black dots). Etna volcano (**b**) is equipped with one small-aperture infrasound array (ETN, black dot) deployed at 5 km from the summit crater. View of Etna volcano from the Catania airport (**c**). Picture taken by E.M. Maps in (**a**,**b**) are created with Matlab R2015B (www.mathworks.com).

Infrasound data used in this work were collected by one small aperture (~200 m) array (ETN, Fig. 1) operated by the University of Firenze, on Mount Etna volcano at a short distance (<6 km) from the summit craters, and three large aperture arrays (>1.5 km), deployed at source-to-receiver distances >500 km (Fig. 1).

The array AMT is a 4-elements array with an aperture of 1.6 km, deployed in central Italy at a distance of 640 km and an azimuth of 336°N from Etna. The array IS48, in Tunisia, is an 8-element array with an aperture of approximately 2 km, located at a distant of 560 km and with an azimuth of 246°N from Etna. The array, OHP, in Southern France, is a 4-elements array with an aperture of 2 km located at 1040 km and with an azimuth of 310°N from Etna. All arrays are equipped with MB2005 microbarometers^25^. While IS48 is part of the IMS network, AMT and OHP were deployed within the ARISE European Project (www.arise.eu).

### Etna volcano and lava fountain events

Etna volcano is the most active and best monitored volcano in Europe (Fig. 1). Its eruptive activity consists both of effusive and explosive eruptions. Explosive activity is generally of mild intensity and characterized by small scale Strombolian explosions from the summit craters (Fig. 1b). This persistent activity is, at times, punctuated by higher energy lava fountains (LF), driving a sustained lava column up to 2000 m height above the vents and feeding ash plumes up to 15 km altitude^26^. Activity renewed in January 2011^11,27^, and is still ongoing with more than 60 LF episodes and ash-rich explosions from different summit craters recorded since then^12^. The last episode occurred on July 19, 2019.

Atmospheric ash injection and tephra fallout during LF episodes can affect nearby cities and airports as well as air traffic. A volcanic ash encounter during a LF event occurred in 2008^3^, immediately after a flight’s departure from Catania airport. The close distance (~30 km) of Catania airport from the summit of Etna volcano (Fig. 1c) requires a very short latency between the occurrence of the eruption and its notification to the airborne flights. Therefore, the automatic recognition and notification of an eruption is a crucial effort to improve volcano monitoring and to support the Italian Civil Protection Agency and civil aviation^12^.

Explosive eruptions at Etna are typically preceded by a clear increase of seismic and infrasound signals^11^, which peak in amplitude during the paroxysmal phase of the event, when the explosive column fully develops and the injection of ash in the atmosphere occurs. Visual observations show that this precursory phase is usually dominated by discrete Strombolian explosions^28^.

Attempts to develop automatic early warning systems for LF at Etna volcano based on seismic tremor, have been carried out since early 2000^29^. However, a more complex unsupervised classification is required to improve its reliability^30^. An operational early warning system was recently realized using an infrasound array at short (5 km) source-to-receiver distance^12^, which is capable of efficiently recognizing the transition between the Strombolian phase and the proper LF event.

### Long range infrasound array observations of etna volcano

Infrasound from eruptive volcanoes can be recorded at large source-to-receiver distances^15–21^, but signal detectability is heavily affected by seasonal and daily stratospheric winds variations^22^. The ability to detect small-size events (VEI 2 or 3), and to infer information on the volcanic source, is thus under debate and still poorly constrained.

**Figure 2:**
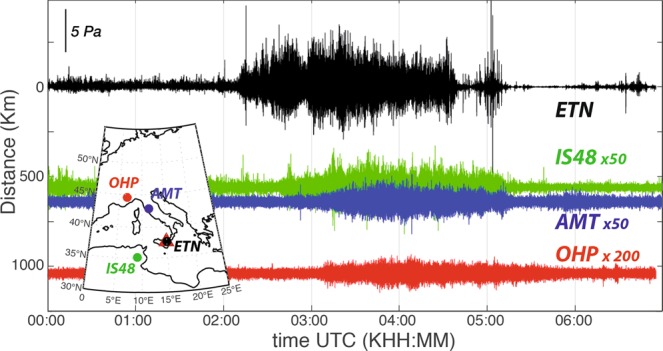
Infrasonic signals from Etna volcano associated to the lava fountain recorded on May 21, 2016 at ETN (black, at 5 km), IS48 (green, at 560 km), AMT (blue, at 640 km) and OHP (red, at 1040 km) infrasound arrays. Local observations are band-pass filtered between 1 and 10 Hz while long-range observations are band-pass filtered between 1 and 3 Hz. Signal amplitudes are scaled to provide sufficient details of the waveforms. The array locations (dots) with respect to Etna volcano (red triangle) are shown in the inlet figure.

We performed an analysis of infrasound observation of eruptions at Etna volcano, considering three large aperture arrays (AMT, OHP, IS48) deployed between 560 and 1040 km from Etna (Fig. 1a). We compare long-range infrasound observations with the near-source records (Fig. 2) by the local infrasound array (ETN) deployed at ~5 km from the summit craters (Fig. 1b).

The efficiency of long-range infrasound monitoring is analyzed during July 2014 and December 2016, when Etna erupted ten times^12^.

#### Range corrected remote observations

Long-range infrasound propagation strongly depends on the vertical profiles of wind and temperature^22^. The variation of sound velocity with height produces a return of infrasound energy from various layers, resulting into a complex infrasound waveform at distal stations. For the specific case of explosions at Etna volcano, stratospheric, mesospheric and thermospheric arrivals at AMT array were reported^31^. The different propagation paths result into different frequency components, with thermospheric arrivals typically lacking the high frequency component due to increased attenuation within the low density, high altitude atmosphere^22^. Infrasound ducting is more efficient within the stratospheric waveguide, between the ground and the stratopause. Stratospheric arrivals are typically characterised by the largest amplitude in the high frequency band (1–3 Hz), where large amplitude of persistent coherent signals, such as microbarom, is absent^32^, and are always recorded as first arrivals at ranges exceeding 200 km from the source.

Therefore, we consider, solely, the frequency band (1–3 Hz) corresponding to stratospheric arrivals to investigate the long-range propagation of signals from volcanic eruptions.

**Figure 3:**
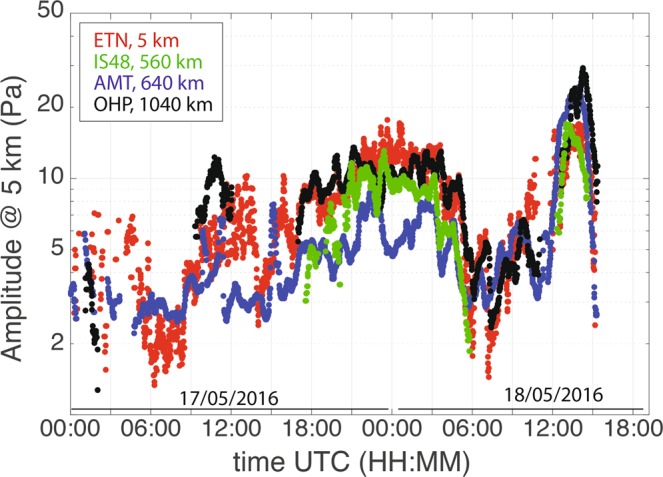
Infrasound amplitudes recorded at ETN array (black dots) between May 17, 2016, 00:00 UTC and May 18, 2016, 18:00 UTC compared with records at IS48 (green dots, at 560 km), AMT (blue dots, at 640 km) and OHP (red dots, @ 1040 km) arrays corrected for attenuation and propagation time.

A methodology that accounts for the attenuation of infrasonic waves, considering both the frequency of the signal and the atmospheric profile along the path, has been developed^33^. This allows for the correction of the amplitude for attenuation along the path and retrieving the pressure at the source (Fig. 3).

Along the propagation paths, we consider a frequency-dependent attenuation relation based on range-independent parabolic equation (PE) simulations^33^, coupled with realistic atmospheric profiles:

1 $${A}_{P}(f,{V}_{eff\_ratio})=\frac{1}{R}{10}^{\frac{\alpha (f)R}{20}}+\frac{{R}^{\beta (f,{V}_{eff\_ratio})}}{1+{10}^{\frac{\delta -R}{\sigma (f)}}}$$

where *R* is the source-to-receiver distance, *f* is the frequency of the signal, and *α, β, δ* and *σ* are parameters derived from multidimensional curve-fitting of PE simulations^33^. The dimensionless parameter (*V*_*eff_ratio*_) is defined as the ratio between the effective sound speed (sound speed added to the along-path wind speed) at 50 km altitude and the sound speed at sea level. *V*_*eff_ratio*_ is averaged along the propagation path^33^.

The first term describes the attenuation of the direct wave by geometrical spreading and exponential decay (*α*). The second term describes the attenuation in the acoustic duct, with *β* being the transmission loss accounting for the geometrical spreading and dissipation of stratospheric and thermospheric waves*, δ* is the width of the shadow zone, and *σ* is a scaling distance controlling the attenuation in the shadow zone^33^.

*V*_*eff_ratio*_ is obtained from temperature and wind profiles provided by the European Centre for Medium-Range Weather Forecasts (ECMWF) operational model. The ECMWF model is based on 137 vertical levels up to 0.01 hPa, with a horizontal resolution of half a degree and a temporal resolution of 6 hours (https://www.ecmwf.int/fr).

According to Eq. (1), refraction of energy in the stratopause region towards the ground is predicted downwind, when *V*_*eff_ratio*_ > 1. On the contrary, variable attenuation, strongly controlled by the frequency of the signal, is expected upwind (*V*_*eff_ratio*_ < 1)^33^. Considering the frequency of the recorded signal (between 0.8 and 2 Hz), the transmission loss for upwind propagation is approximately twice as large as the one expected downwind. The difference between upwind and downwind situation results into events that can only be detected downwind at a given source-to-receiver distance.

This results into a pronounced seasonality of detection capability, with infrasound from sources located East of the array preferentially detected during the summer in the Northern hemisphere and during the winter (austral summer) in the Southern hemisphere. Considering the position of the infrasound arrays used in this study, with respect to Etna volcano, efficient downwind propagation is expected during the summer months (between April and September) while unfavourable upwind propagation prevails during the winter months (between October and March).

The comparison of excess pressure, recorded by infrasound arrays deployed at large distances (up to 1040 km) with local array observations, clearly shows that the pressure time history at the source can be inferred from distant records with a good degree of accuracy. The ratio between the recovered source pressure, once corrected for attenuation using Eq. (1), and source pressure recorded locally, ranges between 0.5 and 2 (Fig. 3) for >80% of data recorded by remote arrays (80% for AMT, 93% for OHP and 92% for IS48).

#### Infrasound parameter derived at regional scale

Infrasound data recorded at the regional arrays (IS48, AMT and OHP) are band-pass-filtered between 1 and 2.5 Hz and processed by applying a multichannel correlation analysis in the time domain, over a 60-second-long time window (*w*) with a time step (*δt*) of 10 seconds. The algorithm of infrasound signal detection, applied here^34^, assumes a plane wave and identifies the signal from Etna volcano in terms of back-azimuth and apparent velocity. Each detection is associated with the corresponding values of time, pressure amplitude, back-azimuth, and apparent velocity.

Infrasound collected from the local array deployed at Etna volcano (ETN) has been processed by applying a grid-search procedure for a source located within the crater area^12^. The algorithm is applied over 5-second-long time windows (*w*) of data, recorded by the local array and shifted by a delay (*δt*) of 1 second.

In order to identify infrasound associated with volcanic activity at Etna, the same procedure used to issue the Early Warning with the local infrasonic array, is applied here to long-range infrasound observations. The operational Early Warning at Etna is based on the infrasonic parameter *IP*^11,12^, which is expressed as:

2 $$IP={N}_{det}\cdot {P}_{m}$$

where *P*_*m*_ is the mean infrasonic pressure and *N*_*det*_ is the number of infrasound detections with a back-azimuth consistent with Etna volcano recorded in a given time window. This is calculated as:

3 $${N}_{det}={\sum }_{i={t}_{o}}^{{t}_{o}+w}i(a{z}_{V}-\delta az < a{z}_{i} < a{z}_{V}+\delta az).$$

where *w* is the duration of the time window used in the calculation (1 minute for the local ETN array^12^) while the index *i* corresponds to detections with a back-azimuth (*az*_*i*_) consistent with the back-azimuth of Etna volcano (*az*_*V*_). The minimum delay time between successive detections satisfying Eq. (3) is equal to *δt*.

Following Eq. (3), detections are filtered according to the back-azimuth of Etna volcano (*az*_*V*_) and allowing a maximum deviation (*δaz*) of +/−3° (i.e. between 126 and 132 °N for OHP, between 148 and 154 °N for AMT, and between 60 and 66 °N for IS48). Such a deviation has been introduced to account for the effects on the acoustic raypath induced by transverse winds observed during the period of analysis. Stronger transverse winds could indeed cause larger deviations of the recorded infrasound back-azimuth^35^.

For the operational warning system of Etna volcano, the parameter *IP* is calculated every minute^12^. *IP* increases with the number of detections per minute (*N*_*det*_) and with excess pressure (*P*_*m*_). Therefore, it is strongly related to the persistence of the infrasound signal. Considering the processing based on the local infrasound array deployed at Etna Volcano^12^ (*w* = 60 s and *δt* = 1 s), *N*_*det*_ reaches a maximum of 60 when the infrasound from Etna is recorded persistently.

For the remote arrays considered here, the parameter *IP* is calculated every minute, by applying Eq. (1) and considering detections from the back-azimuth consistent with Etna volcano, over a time window (*w*) of 20 minutes (Eq. (1)). Such a longer time window is tuned according to the propagation range, as multiple arrivals produced by a single transient explosion would result into a larger number of detections, when the source to receiver distance is increased. For the specific case of Etna volcano, a 20 minute long window (*w*) allows the recording of all possible arrivals at a source-to-receiver distance exceeding 500 km^31^.

Considering a minimum time shift (*δt*) of 10 s and a time window of 20 minutes adopted to calculate *IP*, the *N*_*det*_ at IS48, AMT and OHP arrays, would peak at the maximum value of 120, when the infrasound produced by Etna volcano becomes continuous. The *IP* values depend indeed on the processing parameters (time window and time shift), which account for the travel time, and therefore duration, of the waves.

**Figure 4:**
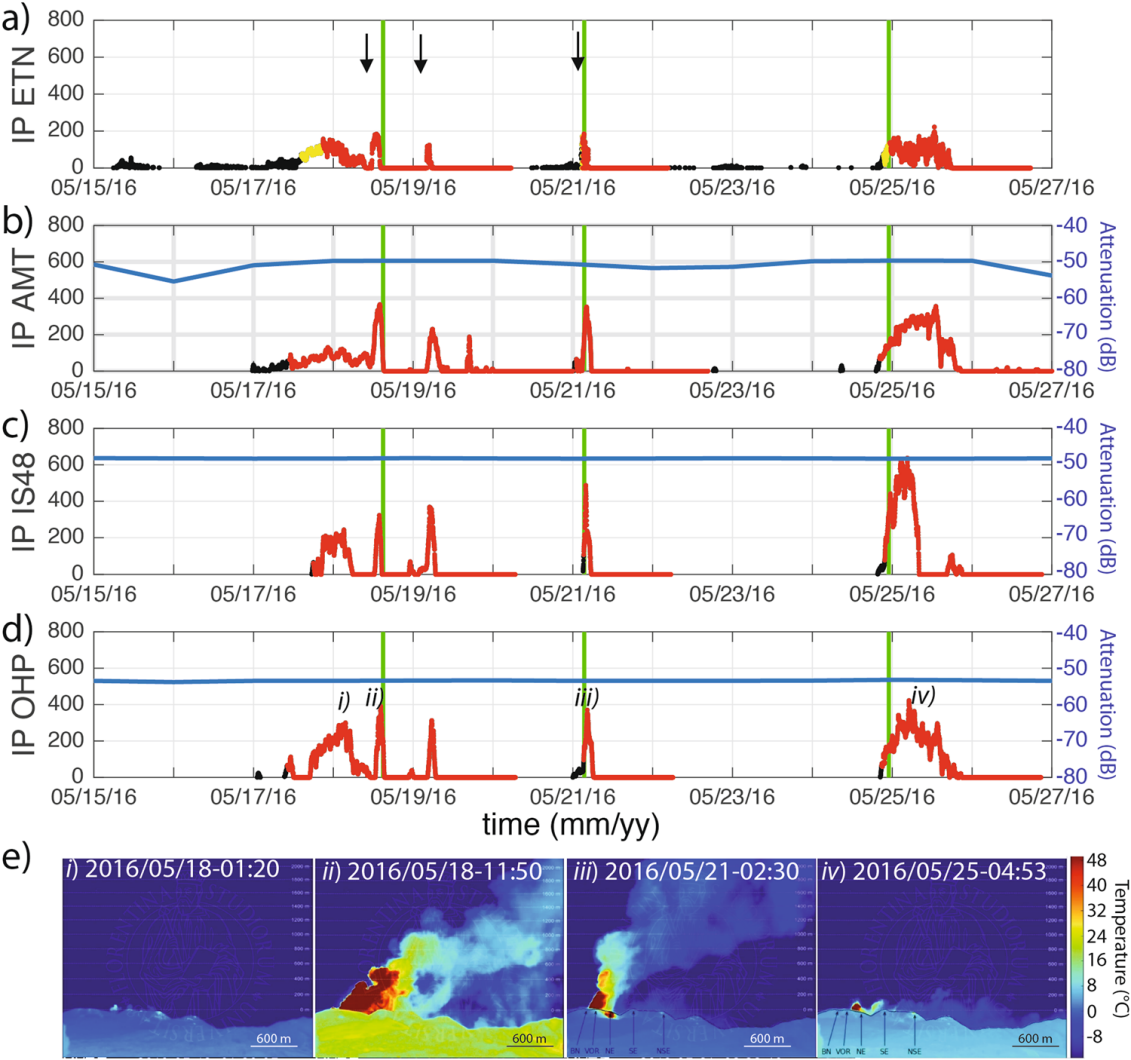
Temporal variations of *IP* (dots) and early warning (EW) status calculated at ETN (**a**), AMT (**b**), IS48 (**c**) and OHP (**d**) arrays for the time period between May 15 and May 27, 2016. Dots are color coded according to the EW status (black – no alert; red – alert). In subplots (**b**), (**c**) and (**d**) the blue line shows the attenuation used to correct the recorded infrasound amplitude. (**e**) Snapshots of a thermal camera deployed on the southern flank of Etna volcano, at an elevation of 2000 m and a distance of 6500 from the summit craters, showing the different eruptive episodes highlighted by labels (i), (ii), (iii) and (iv) in subplot (**c**). The green lines in subplots (**a**–**d**) show the time of issuance of Volcanic Ash Advisories (VAA) by Toulouse VAAC with “red“ aviation color code. The black arrows in subplot (**a**) mark the occurrence the 3 lava fountains.

The mean amplitude of acoustic detections at the receiver (*P*_*m*_) is corrected following Eq. (1) for the attenuation (Fig. 3), while the number of detections per minute (*N*_*det*_) is normalised to the value of 60. This allows the evaluation of an *IP* that is independent of the source-to-receiver distance (Fig. 4).

Despite being of relatively low energy (VEI~2), the eruptive activity at Etna volcano is powerful enough to radiate infrasound at distances exceeding 1000 km. In May 2016, easterly stratospheric winds were creating a stable atmospheric duct for infrasound propagation westward of Etna volcano. This is highlighted by the low attenuation along the source-to-station path (blue lines in Fig. 4b–d), that has very stable values spanning between a minimum of −48 dB, for the closest array (IS48), up to a maximum of −53 dB, for the most distant array (OHP).

### Long-range infrasound early warning

The calculation of the *IP* proposed here, corrected for attenuation and normalized for the number of detections, allows us to use the same thresholds of the parameter *IP* tuned during ten years of infrasonic record at the local array ETN, and used for the early warning system^12^. At ETN, when *IP* exceeds the threshold values for at least five consecutive minutes, the system automatically delivers early warning messages of pre-alert (EW1 when *IP* > 60) and alert (EW2 when *IP* > 120) of a possible ongoing explosive eruption^12^. A previous analysis^12^ clearly showed how the EW1 early warning *IP* > 60 threshold (Fig. 4b–d) had a good percentage of success (96.6%) in anticipating the explosive eruption by almost ~1 hour. For six years, only once was EW1 not associated with a clear lava fountain or ash eruption. This corresponds to ~1.7% of false positive alerts but with no false negative alerts^12^. This threshold was applied here to distinguish between background and eruptive phases at regional and global scales (Fig. 4a).

Therefore, notification of volcanic eruptions using long-range infrasound array observations (LEW), is delivered only when the *IP* exceeds the threshold value of 60, for a minimum of 20 consecutive minutes. This time interval is longer than the five minutes considered for the local array, and accounts for multiple arrivals of the refractions in the atmosphere recorded at large distances, and also in the case of a single transient event. Activity is considered to return back to normal only when *IP* < 8 for a period of 24 hours (Fig. 4).

**Figure 5:**
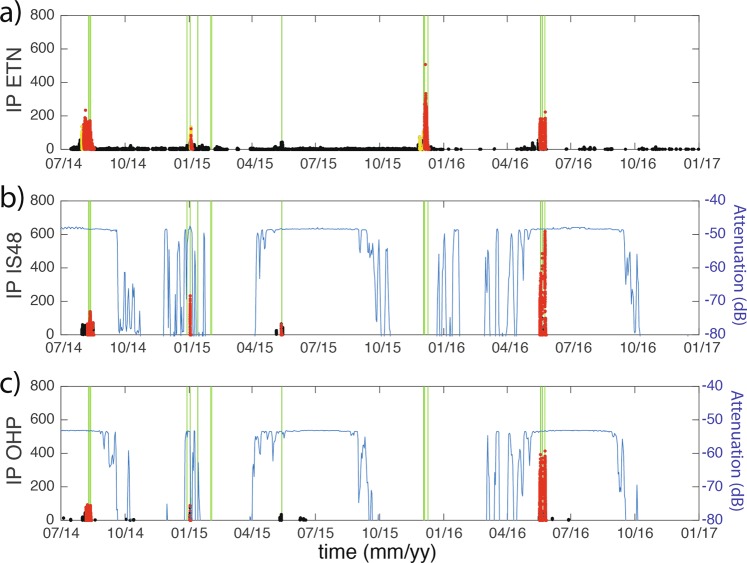
*IP* calculated at ETN (**a**), IS48 (**b**) and OHP (**c**) arrays. *IP* is color-coded according to the activity level inferred from infrasound observations. In (**b**,**c**) the blue line shows the attenuation along the propagation path from Etna volcano to the remote arrays calculated considering ECMWF atmospheric profiles.

The *IP* parameter calculated for the IS48 and OHP arrays, between July 2014 and December 2016, nicely agrees with *IP* calculated at local distance of ~5 km (Fig. 5). As expected, the *IP* reaches high values during summer, while it remains at low values during wintertime, because of the large attenuation (<−60 dB), that prevents infrasound from Etna from efficiently propagating westward.

For this reason, the eruptive phase of December 2015, despite being the most energetic of the period, with ash column reaching about 15 km height^26^, was not detected by the OHP and IS48 arrays. On the contrary, the eruptive episode of January 2, 2015, even if during the wintertime, was well recorded at both IS48 and OHP and marked by a sharp increase of *IP* (Fig. 5b). This unexpected “winter” detection is related to favourable propagation conditions at the time of the eruption, generated by a minor stratospheric warming event in the Arctic, which caused stratospheric wind reversals at the beginning of January 2015^36^.

### Reliability of the infrasound notification

The reliability of a long-range volcano early warning (LEW) system has been checked by comparing the timing of the infrasonic alert with the timing of the VAAs (Volcanic Ash Advisory) issued by Toulouse VAAC between July 2014 and December 2016. For the specific case of Etna volcano, Toulouse VAAC is gathering information from the Istituto Nazionale di Geofisica e Vulcanologia (INGV), that is performing real-time monitoring of Etna volcano with a dense geophysical network, cameras and direct field observations^37,38^. Whenever the activity at Etna volcano increases and might lead to an eruptive phase, INGV issues a Volcano Observatory Notification for Aviation (VONA) to Toulouse VAAC, which eventually issues a VAA.

**Figure 6:**
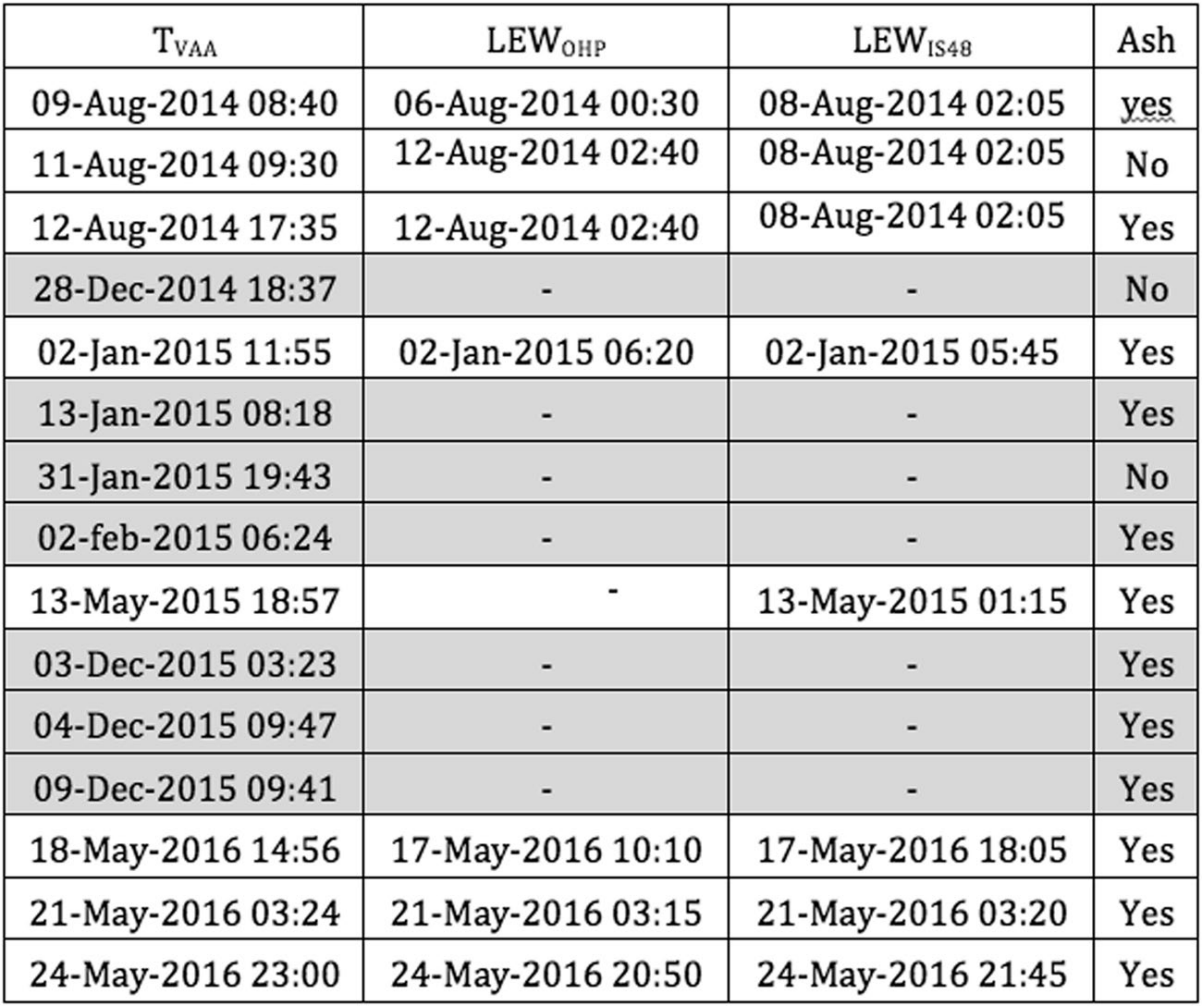
Time of issuance of VAAs by Toulouse VAAC (column 1, T_VAA_); EW issued from infrasound records at OHP (column 2, LEW_OHP_) and IS48 (column 3, LEW_IS48_). Column 4: observed ash injection in the atmosphere. The rows highlighted in grey indicate periods of upwind propagation to the arrays.

Starting from the summer of 2014, VONAs, issued by INGV, include an aviation color code (yellow, orange and red), reflecting the likelihood of having ash dispersed into the atmosphere. The same information is eventually reported in the VAAs, along with information on the ash plume observed by satellite and ash plume extension predicted by models (Fig. 6).

We limited our comparison to VAAs associated with aviation color code “red” warnings, indicating volcanic activity that is most likely expected to be related to significant ash emission into the atmosphere. During the considered time period, Toulouse VAAC issued 52 red color VAAs, most of which, however, indicated the persistency of the volcanic eruption. Therefore, only the timing of the first advisory VAA has been considered and the successive VAAs, if issued less than 24 hours apart from each other, have been disregarded. This limits the current comparison to 15 VAAs that are compatible with 15 distinct eruptive episodes (Fig. 6).

Whenever it is observed, the presence of ash in the atmosphere is reported in Fig. 6. Infrasound notifications delivered by the OHP array represent 47% of success, with seven events detected out of the 15 eruptions, whereas, the IS48 array has a slightly larger success of 53%, with eight out of 15 eruptive episodes clearly detected (Figs. 5 and 6).

This low percentage of success is, in reality, reflecting the control of variable stratospheric winds on acoustic propagation. For upwind situation, the signal attenuation is significant (Eq. 1) and the resulting array detection capability becomes poorer (Fig. 5). With an attenuation lower than <60 dB (Fig. 5), the detectable eruptions reduce to eight episodes (Fig. 6). In this case, the OHP array is detecting seven out of the eight events, resulting into an efficiency of 87%. The efficiency increases to 100% at the IS48 array during downwind propagation conditions. No eruptive episodes have been detected by both arrays during upwind propagation conditions (Fig. 6).

It is worth noting that the methodology presented here is systematically removing other possible sources of infrasound originating from the same back-azimuth of Etna volcano during the 2.5-year-long period of analysis and no negative false alerts have been issued. Even if the proposed methodology performed well during the studied time period, further evaluation should be pursued by considering other volcanoes, as persistent sources of infrasound located along the source-to-receiver travel path (such as microbarom), or at close distance from the array, might result into false alerts and limit its efficiency. Additionally, because the proposed methodology is based mostly on stratospheric arrivals, its efficiency is strongly time dependent (Fig. 5) and depends on the correct forecasts of stratospheric winds.

**Figure 7:**
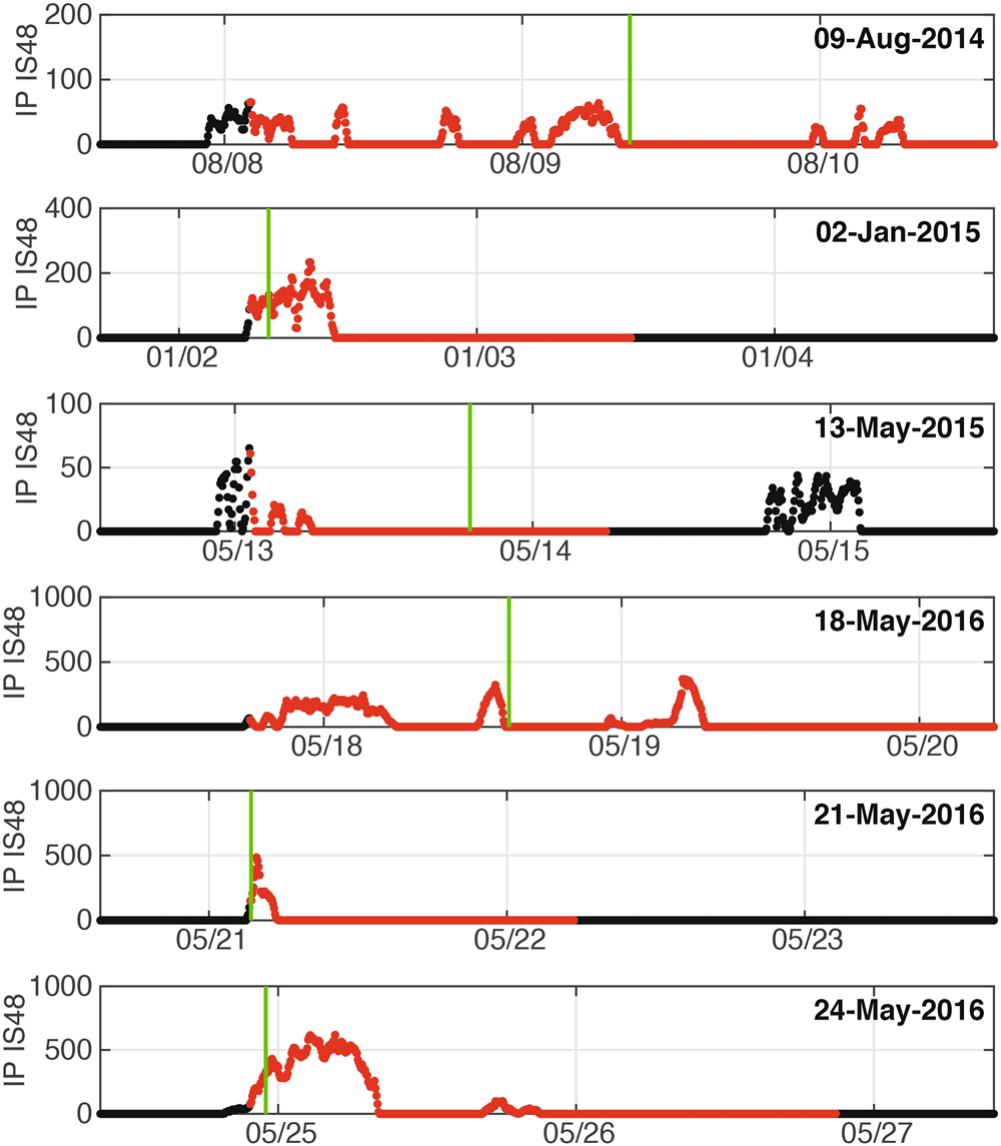
Detail of *IP* calculated at IS48 for 6 VAAs issued during period of favourable propagation conditions (Fig. 6). *IP* is color-coded according to the activity level inferred from infrasound observations. The green vertical lines show the time of issuance of Volcanic Ash Advisories (VAA) by Toulouse VAAC with “red“ aviation color code.

Despite the pressure wave requiring approximately 31 minutes to propagate from Etna to IS48, and about one hour to OHP, the explosive activity would have always been detected before a VAA issued by the Toulouse VAAC (Figs. 4, 6 and 7). Considering that Etna is one of the best monitored volcanoes in the world, this result strongly supports the use of infrasound arrays, at regional distances, to issue automatic notifications of ongoing eruptions.

## Discussion

In our global society, a prompt response to volcanic eruptions is required to mitigate the impact of the volcanic hazard on aviation. Many volcanoes worldwide are poorly monitored, or still unmonitored, and most of the time notifications on volcanic eruptions are reported, mainly after satellite observations or by visual observation made by the aeroplane pilots, after (sometime hours) the event has occurred^39^. Thus, the monitoring of volcanic activity in real time and on a global scale, is thus one of the mandatory issues to be challenged in the next decades.

We have shown that infrasound observations of eruptive activity at Etna volcano, recorded at global distances (between 560 and 1040 km), can be used to provide an early warning system for explosive eruptions. The method proposed is based on the same infrasound parameter (*IP*) successfully used to provide automatic early warnings of explosive eruptions with local (~5 km) infrasonic arrays^12^. The infrasonic parameter *IP* is calculated as the average of the acoustic amplitude, times the rate of detections in a given time interval^11,12^.

The use of a frequency-dependent semi-empirical attenuation relation^33^, coupled with realistic atmospheric profiles, allows the retrieval of the source pressure with amplitude ranging between 0.5 and 2 times the one recorded locally.

Under downwind situation, infrasound propagates efficiently in the stratospheric waveguide and remote observations are comparable to observations at local range (<tens of km). Correcting the signal amplitude measured at global range allows applying the same *IP* threshold used at local range.

During favourable downwind propagation conditions, the *IP* calculated at OHP and IS48 arrays, has resulted into a success rate of more than 87% in detecting explosive eruptions at Etna (100% for IS48 at 560 km), without false alarms.

Despite of the latency due to the propagation time (~30 minutes), infrasound-based notification at the IS48 and OHP arrays precedes by almost 10 hours (Fig. 7) the corresponding VAAs message issued by the VAAC (Fig. 6).

Given the low intensity of eruptive activity at Etna (~VEI2), and the performance of the proposed methodology, the presented results suggest that this infrasonic-based procedure might be successfully applied to other volcanoes worldwide. As recording distance is generally increasing with the energy of the eruption, infrasound from eruptive volcanoes can indeed be recorded at source-to-receiver distances exceeding 10.000 km^17^. This opens new perspectives in volcano monitoring on a global scale and could represent, in the future, an efficient tool in supporting the VAACs activity.

However, the application of the proposed methodology might be limited by the presence of other persistent sources of infrasound along the volcano-to-received path, such as microbarom, or industrial activity at close distance to the array^21^. Therefore, the use of the proposed methodology needs to be carefully evaluated for each single volcano of interest before application, by identifying possible sources of false alerts.

## Data Availability

The infrasound detections obtained from AMT, OHP and IS48 infrasound arrays between July, 1, 2014 and January, 1, 2017 are freely available in the Open Science Framework repository (https://osf.io/vn7py/).
